# The transfer and transformation of collective network information in gene-matched networks

**DOI:** 10.1038/srep14984

**Published:** 2015-10-09

**Authors:** Takashi Kitsukawa, Takeshi Yagi

**Affiliations:** 1 Graduate School of Frontier Biosciences, Osaka University, 1-3 Yamada-oka, Suita, Osaka 565-0871, Japan

## Abstract

Networks, such as the human society network, social and professional networks, and biological system networks, contain vast amounts of information. Information signals in networks are distributed over nodes and transmitted through intricately wired links, making the transfer and transformation of such information difficult to follow. Here we introduce a novel method for describing network information and its transfer using a model network, the Gene-matched network (GMN), in which nodes (neurons) possess attributes (genes). In the GMN, nodes are connected according to their expression of common genes. Because neurons have multiple genes, the GMN is cluster-rich. We show that, in the GMN, information transfer and transformation were controlled systematically, according to the activity level of the network. Furthermore, information transfer and transformation could be traced numerically with a vector using genes expressed in the activated neurons, the active-gene array, which was used to assess the relative activity among overlapping neuronal groups. Interestingly, this coding style closely resembles the cell-assembly neural coding theory. The method introduced here could be applied to many real-world networks, since many systems, including human society and various biological systems, can be represented as a network of this type.

Many systems can be represented as a network consisting of nodes connected by links^1^,^2^,^3^. Examples include social networks^4^,^5^,^6^ such as acquaintance networks^7^ and collaboration networks^8^, biological networks such as neural networks^6^, food webs^9^, and metabolic networks^10^,^11^, and technological networks such as the Internet^12^ and the World Wide Web^13^. The existence of a link between nodes indicates that interaction or signaling can occur between the nodes. These signals, such as the behaviors of persons in a social network^14^ or neuronal activity in a neural network^15^,^16^,^17^,^18^, transmitted among nodes through links, shape the collective information in the network. Thus, the form of the network information is dependent on how the signals are transmitted in the network, which is subject to the architecture of the links^14^,^19^,^20^,^21^. Links are often made according to local rules, such as the attributes or labels of nodes. For example, people who possess a common hobby have an increased probability of being acquainted, and proteins engaging in a common biological process have an increased probability of functioning together. Such local rules hidden in the creation of links may determine the architecture of the network and thus the network information shaped by the architecture. Nodes often possess multiple attributes or labels, the assembly of which can influence link formation. To analyze the effect of multiple latent node attributes on link formation and network structure, Kim and Leskovec developed the latent multi-group membership graph (LMMG) model, in which nodes are assigned multiple attributes that influence link formation^22^,^23^,^24^. They showed that their model could explain the structure of real networks; however, they did not analyze the effect of multiple node attributes on signal transmission or information transfer.

In this study, we focused on the attributes of nodes that influence link formation, and describe the transfer and transformation of network information using these attributes. For this purpose, we established a model neural network called the gene matched network (GMN), which is comprised of nodes (neurons) that possess attributes (genes).

## Results

### The architecture of the GMN

**Figure 1:**
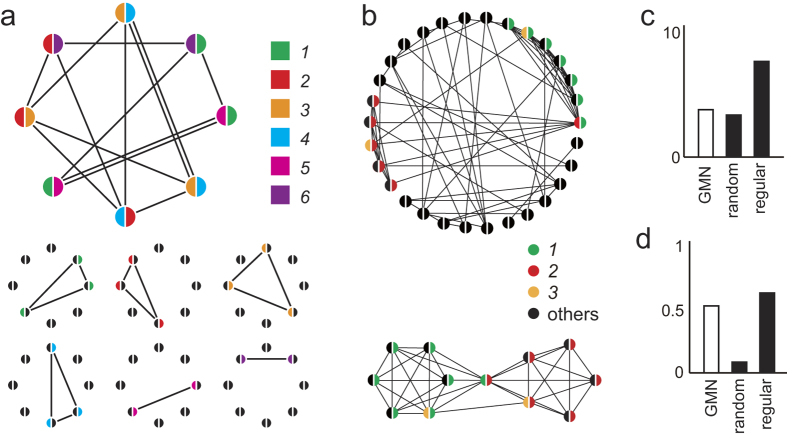
Characteristics of the circular GMN. (**a**) A GMN with eight neurons, each of which expressed two genes from a repertoire of six genes (colors). Neurons expressing common gene(s) were connected. Subnetworks connected by a single gene are shown below. A subnetwork connected by a single gene expressed in three neurons formed a triangle or cluster (genes *1*–*4*). (**b**) A GMN with 30 neurons, each of which randomly expressed 2 genes from a repertoire of 30 genes. Neurons expressing gene *1* (green) and gene *2* (red) were re-aligned and extracted from the GMN (below). Shortcuts made by neurons expressing both gene *1* and *2* or neurons expressing gene *3* (yellow) are shown. (**c,d**) The average shortest path length (**c**) and clustering coefficients (**d**) of a circular GMN with 100 neurons (GR = 50, GE = 5, open bars), a random network, and a regular network (lattice as in Watts and Strogatz, 1998). In the GMN used for c and d, one connection was defined even when a pair of neurons shared multiple common genes.

**Figure 2:**
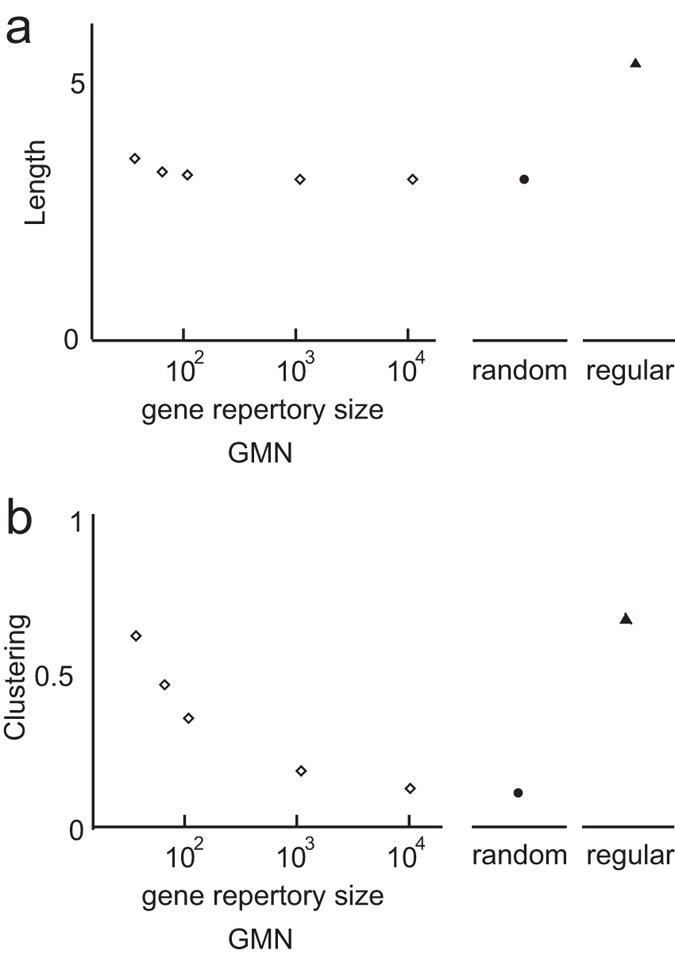
Effect of GR size on the architecture of the circular GMN. Effect of GR on the average shortest path length (**a**) **a**nd the clustering coefficient (**b**) of circular GMNs (open diamonds), random networks (closed circles), and regular networks (with lattices as in Watts and Strogatz, 1998, closed triangles). All of the networks in a and b were circular networks with 100 neurons and 500 undirected connections. Data points were determined from 100 realizations of the network.

In the GMN, each neuron expresses genes that are randomly selected from a gene repertoire (GR), and neurons expressing any common genes are connected, forming subnetworks (Fig. 1a,b). The neurons express multiple genes (a GMN with two genes is shown in Fig. 1), and each neuron belongs to as many complete subnetworks as the number of genes it expresses. This overlapping feature of the GMN contributes to the generation of shortcuts between non-adjacent neurons (Fig. 1b). Thus, the GMN is rich in clusters and shortcuts. We found that a GMN consisting of 100 neurons, each expressing two genes (GE = 5), with a GR of 50 genes (GR = 50), exhibited the characteristics of a small-world network^6^ (Fig. 1c,d). Next, we analyzed the effect of the GR size on the GMN’s network properties. While the shortest path length of the GMNs at any GR showed little difference from that of a random network (Fig. 2a), the clustering coefficients of the GMNs with small GRs (GR = 20, 50) were much higher than that of the random network, indicating that the GMNs with small GRs were small-world networks (Fig. 2b).

### Reliable transfer of network information in the GMN

**Figure 3:**
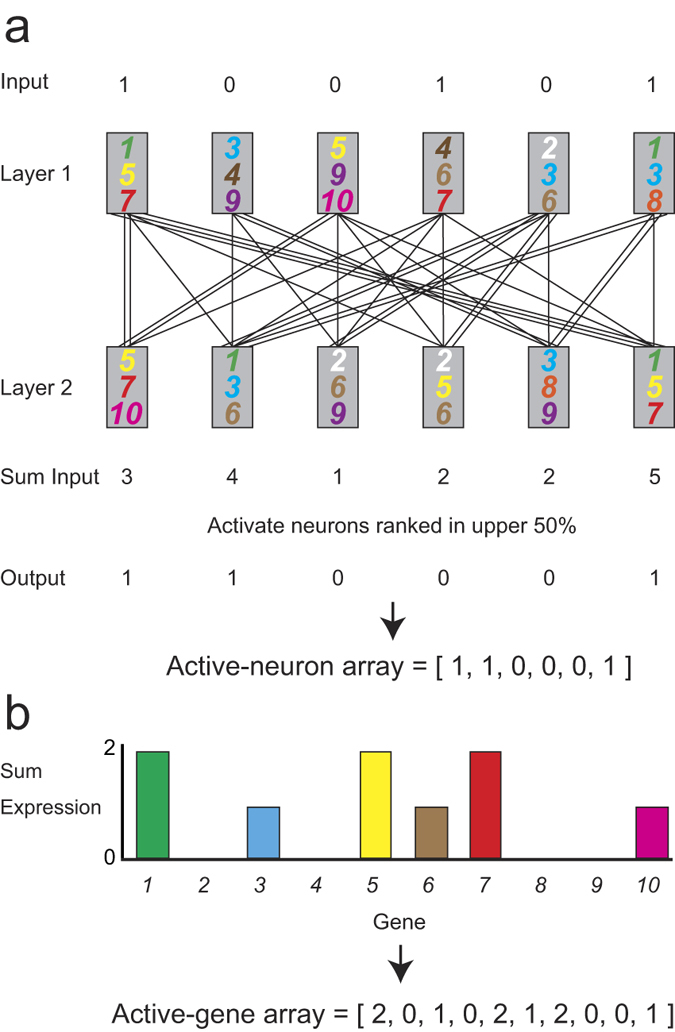
Scheme of information transfer in a multi-layer GMN. (**a**) A GMN with two layers of neurons, each expressing 3 genes from a repertoire of 10 genes. When a neuron in layer 1 is activated (input = 1), the signal are multiplied by the number of connections and transmitted to layer 2 neurons. The input to each layer 2 neuron is the sum of inputs (Sum input) from activated neurons in layer 1. In this instance, the neurons whose summed inputs rank in the upper 50% (AP = 50) are activated, representing the active-neuron array. (**b**) Histogram of genes expressed in the activated neurons, representing the active-gene array.

Clusters are reported to influence the spreading of behaviors and diseases^14^,^21^, implying that clusters have the potential to store information transmitted by recurrent signals. Thus, the overlapping of complete subnetworks, a characteristic of the GMN, was predicted to influence information flow. To study information transfer in the GMN, we analyzed multi-layer GMNs. In general, information cannot be reliably transferred in randomly connected multi-layer networks, because the signals impinging on each node converge and diverge randomly as they move through the layers, which is equivalent to averaging (Supplementary Fig. 1a). In contrast, when signal transmission follows the architecture of the GMN, the complete subnetworks in the GMN, each of which consists of neurons expressing a particular gene (Supplementary Fig. 1b), may function to store, integrate, and convey information to the next layer. If this view is correct, multi-layer GMNs with large complete subnetworks, due to a relatively small GR (such as 20 or 50) (see Figs 1b and 2b; high clustering coefficients reflect large complete subnetworks) should show reliable signal transfer. To test this hypothesis, we analyzed the signal transmission obtained using a multi-layer GMN and a degree-matched random network. In this experiment, signals are transmitted in a top-down manner through connections between two neuronal layers (Fig. 3a). The signals are summed in the second layer, and the activated neurons in this layer are determined according to the summed input of each neuron and the activation percentage (AP) of the layer. The AP is the percentage of neurons being activated in a particular layer. The profile of activated neurons is represented by the active-neuron array (Fig. 3a), and the profile of genes expressed in the activated neurons is represented by the active-gene array (Fig. 3b). If the hypothesis is correct, the active-gene array should carry information and serve as a readout of information transfer (See Discussion).

**Figure 4:**
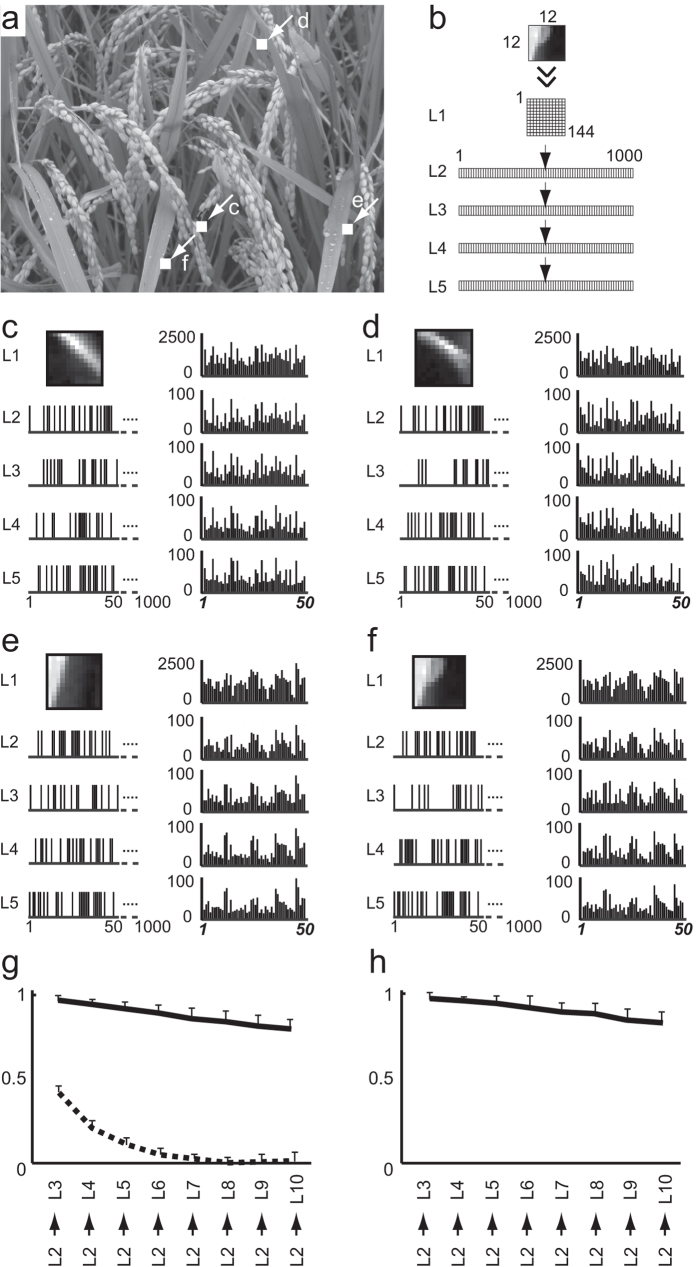
Transfer of image information in a GMN. (**a**) Photograph (553 × 737 pixels) from which small image inputs (12 × 12 pixels) were randomly selected (1000 examples of the small images are shown in Supplementary Fig. 2). Locations of the four small image inputs shown in (**c–f**) are indicated by arrows. (**b**) Architecture of the GMN (GR = 50, GE = 5, AP = 50). Each layer contained 1000 neurons except for the first layer (144 neurons). In layer 1, each neuron received input, the value of which corresponded to the pixels in the small images. (**c–f**) Input images and the resulting active neuron patterns (left). (The first 50 of 1000 neurons in each layer are shown.) Histograms of the active-gene arrays in layers 1–5 are shown (right). Note that similar images (**c–f**) had similar active-gene arrays. (**g**,**h**) Linear regression analysis of the cross-layer consistencies from layer 2 to each of the following layers, calculated using the active-neuron arrays of the GMN (solid line in (**g**)) and a random network (dashed line in (**g**)), and using the active-gene arrays of the GMN (**h**). Data shown are the means ± s.d., n = 10.

**Figure 5:**
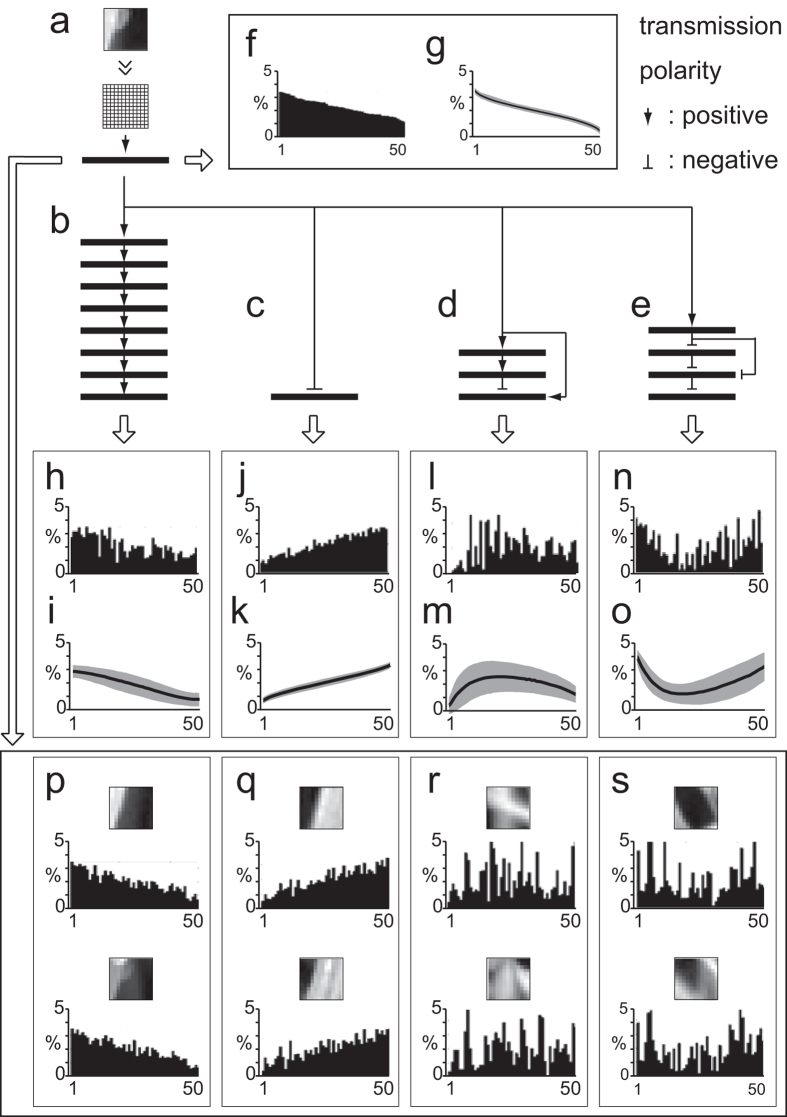
Transformation of active-gene arrays by various GMNs. Various GMNs (GR = 50, GE = 5) were used. Transmission polarities are specified by arrows (positive) and bars (negative). (**a**) Template im**a**ge and common layers (1 and 2). AP (layer 2) = 50%. (**b**) AP (layers 3-10) = 50%. (**c**) AP (layer 3) = 50%. (**d**) GMN representing the cerebellar network, with layers 2-5 corresponding to the pontine nuclei, granule cell layer, Purkinje cell layer, and cerebellar nuclei, respectively. AP (layers 3-5) = 25%. The combination ratio between main and side branches was 1:2. (**e**) GMN representing the basal ganglia, with layers 3-6 corresponding to the striatum, external globus pallidus, substantia nigra pars reticulata, and thalamic nuclei, respectively. AP (layers 3, 4, 5, 6) = 25, 10, 25, 25%, respectively. The combination ratio between main and side branches was 1:2. (**f**) The active-gene array of layer 2, aligned in descending order, called the input order. (**g**) The active-gene array in layer 2 averaged over 5,000 candidate images (s.d., gray area). (**h**,**j**,**l**,**n**) The active-gene arrays at the last layer of GMNs (**b,c,d,e**) respectively. (**i**,**k**,**m**,**o**) The active-gene arrays averaged over the 5,000 images at the last layer of GMNs (**b,c,d,e**) respectively (average, solid line; s.d., gray area). (**p**–**s**) Sample images li**s**ted according to the similarity of their active-gene array at layer 2 to the active-gene array of the template image in the last layer of GMNs (**b,c,d,e**) respectively. The active-gene arrays shown in h-s were re-aligned in the input order.

To explore the functionality of the model, graphical images (12 × 12 pixels, Supplementary Fig. 2) randomly selected from a photograph (Fig. 4a) were used as input signals in the GMN (GR = 50, GE = 5). Figure 4c–f shows four example images and the corresponding responses in each layer with an AP of 50%. Note that similar image inputs (Fig. 4c–f) yielded similar active-gene arrays at each layer. Conversely, similar images could be found by searching images according to the similarity of their active-gene arrays (Supplementary Fig. 3c). Using an AP of 50%, the active-gene arrays of each image showed similar patterns at each of the five layers examined (Fig. 4c–f, right), while the active-neuron arrays were different in each layer (Fig. 4c–f, left). Notably, even when the active-gene arrays at layer 10 (template images) were compared to those at layer 2 (candidate images), similar images were observed (Fig. 5p, Supplementary Fig. 3f and Supplementary Fig. 4p). These results suggest that the active-gene array may be broadly applicable as a representation of network information.

To examine the reliability of information transfer, we analyzed the cross-layer consistency of the distance between a pair of inputs as they traveled across layers 2 through 10 in the GMN. If the information transfer was reliable, the distance between the input signal pairs should be positively correlated with the distance between the output pairs. In other words, similar inputs in a given layer should give rise to similar outputs in the following layers, while dissimilar inputs should give rise to dissimilar outputs (Supplementary Fig. 5). We calculated the cross-layer consistency of the active-neuron arrays and the active-gene arrays in the GMN (GR = 50, GE = 5) and found that both remained high even after passing across 8 layers (Fig. 4g,h and Supplementary Fig. 6). In contrast, the cross-layer consistency of the active-neuron arrays was lost after passing through several layers of a randomly connected network. Thus, the structure of the GMN may serve to maintain the cross-layer consistency.

**Figure 6:**
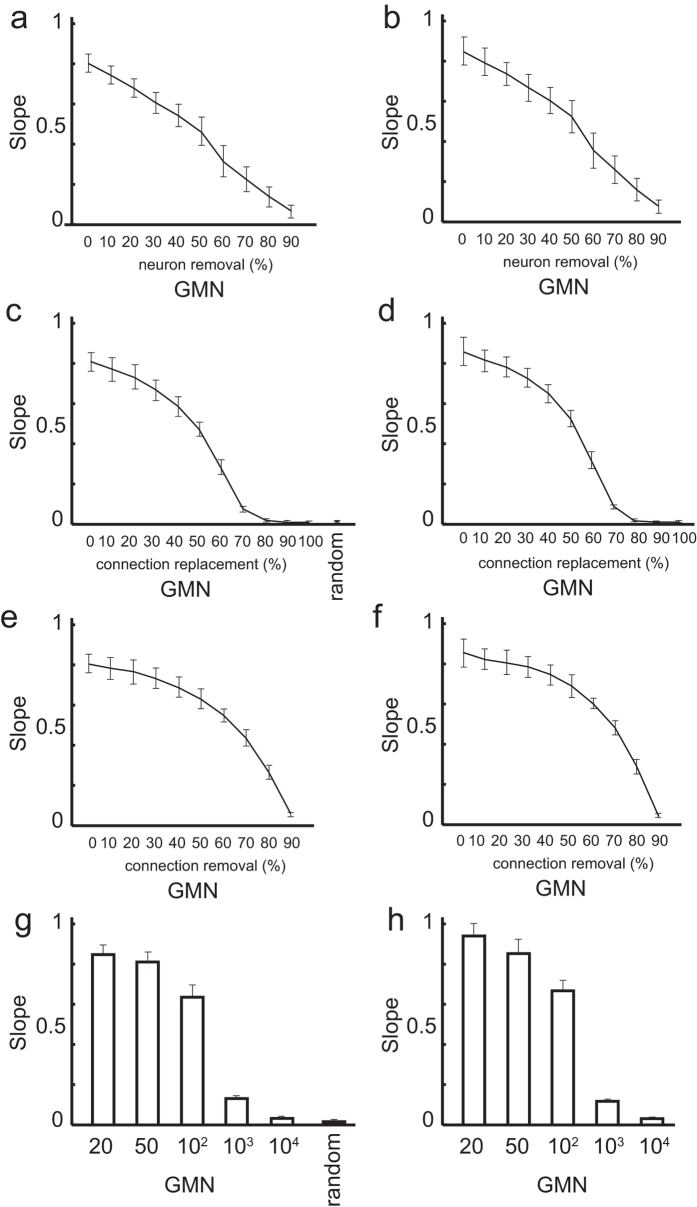
Effect of network deterioration and GR size on information transfer. Slopes were obtained by linear regression analysis of the cross-layer consistencies from layers 2 to 10, calculated using the active-neuron arrays (**a,c,e,g**) and active-gene arrays (**b,d,f,h**). Averages and error bars (s.d.) were calculated over 10 realizations of the network. The input images and the GMN architecture were the same as those shown in Fig. 4, except for the parameters mentioned below. (**a**,**b**) Cross-layer consistency of the GMNs (GR = 50, GE = 5, AP = 50%), in which 0–90% of the neurons were randomly removed. (**c**,**d**) Cross-layer consistency of the GMNs (GR = 50, GE = 5, AP = 50%) in which 0–100% of the connections were randomly replaced, and of the connection-number-matched random connected network. (**e**,**f**) Cross-layer consistency of the GMNs (GR = 50, GE = 5, AP = 50%), in which 0–90% of the connections were randomly removed. (**g**,**h**) Cross-layer consistency of GMNs (AP = 50) with different GRs.

To obtain further insight into how the structure of the GMN maintains the cross-layer consistency, the robustness of the cross-layer consistency was analyzed as the GMN was disrupted by neuron removal, or by random connection replacement or removal. First, we analyzed the effect of neuron removal by randomly removing 10% to 90% of the neurons from the same original GMN. Gradual deterioration of the cross-layer consistency was observed (Fig. 6a,b). Next, we analyzed the effect of connection replacement by randomly replacing 10% to 90% of the connections from an original GMN with 1000 neurons (GR = 50, GE = 5, AP = 50%). Analysis revealed that the cross-layer consistency from layer 2 to layer 10 gradually decreased as more connections were replaced (Fig. 6c,d). Interestingly, in contrast to the neuron removal, a sharp drop in consistency after slow deterioration was observed when 50% of the connections were replaced, indicating that the architecture of the GMN may serve to maintain the cross-layer consistency. A similar pattern of cross-layer consistency degradation was observed when the connections in GMNs were randomly removed (Fig. 6e,f). These results suggest that a loss or error in GMN connections has a moderate effect on the reliability of signal transfer.

We also examined the effect of the GR size (GR = 20, 50, 100, 1,000, 10,000) on the cross-layer consistency of the GMN. To obtain GMNs with comparable numbers of connections, the number of genes expressed (GE) was set to 3, 5, 7, 22, or 71 for a GR size of 20, 50, 100, 1,000, or 10,000, respectively. The number of neurons comprising a subnetwork in a layer was 150, 100, 70, 22, and 7.1 on average, respectively. We found that the cross-layer consistency deteriorated in the GMNs containing large GRs (>1000, Fig. 6g,h); in these cases, signal re-entry into a complete subnetwork would be unlikely due to the small subnetwork size. These findings support the notion that complete subnetworks function to store and convey information. Since the size of the subnetworks would be greater when a layer has more neurons, layered GMNs with large numbers of neurons would exhibit more reliable cross-layer consistency in large GR size conditions.

Finally, we analyzed the response specificity of individual neurons. For every neuron, the images that activated it were averaged, generating the optimum image for that neuron. The averaged images obtained from the neurons of the GMN (Supplementary Fig. 7a) and the random network (Supplementary Fig. 7c) showed almost the same sharp contrast after passing through one layer. Remarkably, the averaged images obtained from the GMN at layer 10 still had sharp contrast (Supplementary Fig. 7b), while the averaged images obtained from the random network became almost uniformly gray (Supplementary Fig. 7d). This result indicates that neurons in the GMN maintained their response specificity in the deep layers, while neurons in the random network did not, due to the random mixing of information (Supplementary Fig. 1). Thus, in the GMN, it is possible to describe input images based on the combination of neurons, which means that the GMN layers can maintain the information of input images.

### Consistent transformation of network information by the GMN

**Figure 7:**
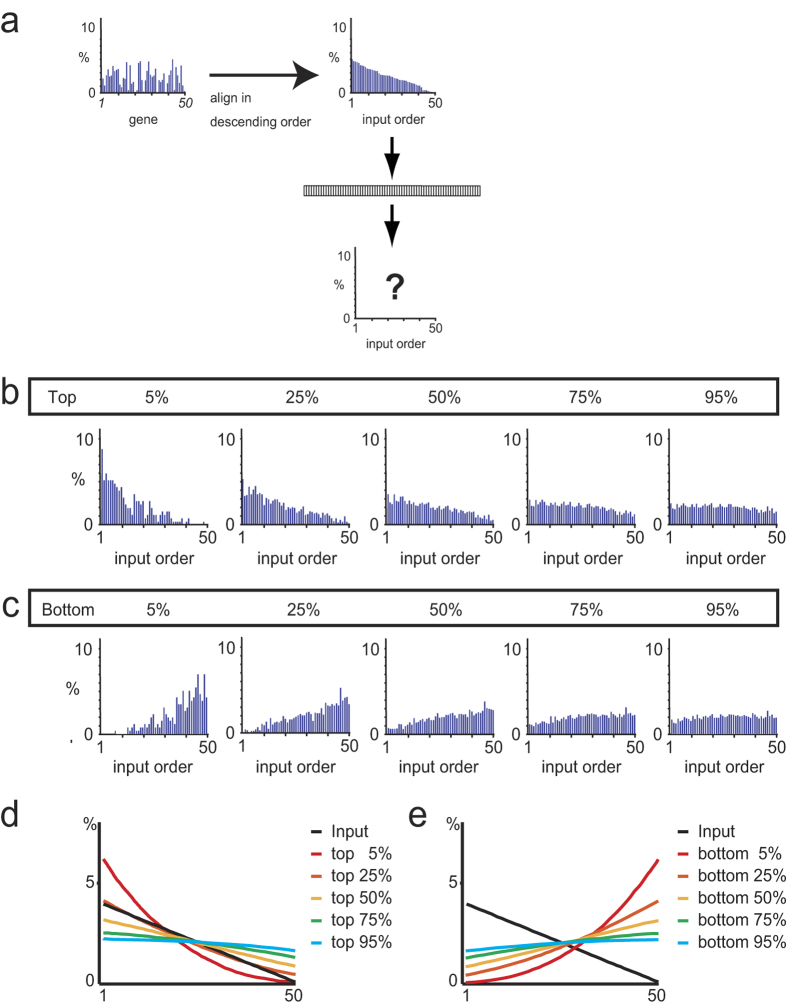
Effect of the AP on the active-gene array in a single layer of the GMN. (**a**) An active-gene array randomly generated for input use (left) and the same array re-aligned in descending order. The order of the genes in the re-aligned array is called the input order. (**b**,**c**) Active-gene arrays obtained after passing through a GMN layer (GR = 50, GE = 5) with an AP of 5, 25, 50, 75, and 95%, when the transmission polarity was positive (**b**) or negative (**c**). (**d**,**e**) Effect of AP on active-gene arrays passing through a GMN layer, using 1000 random inputs, and averaged over 10 independent GMNs, when the transmission polarity was positive (**d**) or negative (**e**).

In the above analyses, the AP was set to 50%, which resulted in reliable signal transmission that could be explicitly observed in the pattern of the active-gene arrays (Fig. 4c–f, right). When the AP was more restrictive (5% and 25%), the expression of high-ranking genes was augmented, while that of lower-ranking genes was diminished (Fig. 7b). In contrast, when the transmission polarity was negative, representing the case in which neurons in the preceding layer were inhibitory neurons, the expression of low-ranking genes was augmented (Fig. 7c). In addition, more complex transformation of the active-gene arrays was achieved by signal bifurcation, and by changing the transmission polarity and AP settings (Fig. 5d,e), for example, which were modeled based on the excitatory and inhibitory connections following those in the cerebellum and basal ganglia, respectively. Notably, the GMN layers were connected on the basis of common gene expression, without incorporating any learning steps.

If the active-gene array represented the information in the images, then transformed active-gene arrays should be reflected in the images in consistent ways. When similar active-gene arrays at layer 2 were searched using the active-gene arrays transformed by negative connection (activation of bottom-ranked neurons, Fig. 5c,j,k and Supplementary Fig. 4c,j,k), negative images of the original images were obtained (Fig. 5q, Supplementary Fig. 4q and Supplementary Fig. 8b). Similarly, image searches were executed using the active-gene arrays transformed by two complex GMNs (Fig. 5d,l,m,e,n,o). These complex GMNs yielded neither similar nor negative images of the original, but many of the resulting images shared common features with each other (Fig. 5r,s Supplementary Fig. 4r,s and Supplementary Fig. 8c,d). Notably, the active-gene array exhibiting augmentation on both ends (Fig. 5n,o) picked up images with two white areas on the right and left sides (Fig. 5s and Supplementary Fig. 8d left), which were observed separately in in the simple GMNs in Fig. 5p,q, respectively. These results indicate that the transformation of image information was consistent with the transformation of active-gene arrays by the non-linear activation of neurons in the GMNs.

## Discussion

Here we established a network model, the GMN, in which node attributes (genes) influence link formation, that can be used to describe the transfer and transformation of network information. We showed that the GMNs with small GRs exhibited the characteristics of a small-world network, including the co-existence of shortcuts and clusters, when the connection numbers were small. In the GMN, the number of complete subnetworks is equivalent to the GR size, which contributes to its high cluster coefficients. Each neuron belongs to as many complete subnetworks as the number of genes it expresses. This overlapping of complete subnetworks causes the GMN to have a short path length.

We found that the multi-layer GMN faithfully transferred information across multiple layers, while information was lost in randomly connected networks. We also found that information transfer in the GMN could be followed by assessing either the active-neuron array or the active-gene array. Since the active-neuron and active-gene arrays can be viewed as two different coding styles, we have designated them as the neuron code and the gene code, respectively.

How do the genes expressed in activated neurons encode information? In the multi-layer GMN, each activated neuron transmits its signal to all the neurons in the next layer that share one or more genes; the neurons in the next layer receive more inputs when they express genes that are frequently expressed in the active neurons in the previous layer. Thus, neuronal activation in this system depends on the genes expressed in the neurons. Given that neurons expressing a common gene form a complete subnetwork in the GMN, neuronal activation can be viewed as the assignment of an activity (or distribution of a unit of information) to each complete subnetwork. Once a unit of information is assigned to a subnetwork it can remain there, because of the re-entry of signals into the subnetwork. This notion is supported by our finding that GMNs with a large GR failed to transfer information reliably. These GMNs contained a large number of complete subnetworks, each of which was composed of a small number of neurons, suggesting that signal re-entry was unlikely and that signal mixture with other subnetworks was more likely. Since each subnetwork is composed of neurons expressing a common gene, the number of subnetworks is equivalent to the GR size. Thus, the information in a GMN can be represented as an array of information units, each of which corresponds to a different subnetwork, which is the active-gene array. The partial removal or replacement of connections had little effect on the reliability of information in the GMN, suggesting that the proportion of activated genes was not affected even with moderate (<30%) flaws in connections.

Our findings suggest that the reliability of information transfer depends on how the node attributes, which are genes in the GMN, determine connections. In the GMN, the number of connections between a pair of neurons is proportional to the number of common genes in the neurons. This linearity probably explains, at least in part, the existence of subnetworks that can transfer information without deformation. Networks with a different connection-attribute contingency, such as the parametric LMMG model developed by Kim and Leskovec^22^,^23^, may have a different condition for information transfer. In the model, nodes are assigned multiple attributes, and the probability of forming links is high when identical attributes are shared between nodes, similar to the GMN, but the connection probability is not linear when multiple attributes are matched between nodes; rather, the connection probability is a product of the probability of each matched attribute. Although information transfer was not analyzed with the model, the transmission by layers would cause distinct effects rather than the linear transmission observed in the GMN.

The present study showed that information was transferred or transformed reliably by the GMN, as demonstrated by the similarity of active-gene array inputs and outputs. Since the percentage of activated neurons is determined by the AP, the signal input into activated neurons is uniformly transmitted, while the signal input into non-activated neurons is discarded. Thus, GMN layers associated with various APs can be considered as nonlinear filters. Surprisingly, information was faithfully transmitted across multiple layers of the GMN under these nonlinear conditions. In contrast, under linear transmission conditions (i.e., when the neuronal input and output were proportional), the activity levels of neurons became similar to each other after signals were passed through a layer, making it difficult to analyze and process the information. Thus, nonlinear transmission, controlled by various APs, contributes to differential neuronal activation, which is required for the effective use of the GMN model.

Highly restrictive AP conditions, with either positive or negative polarity, resulted in increased expression of the top- or bottom-ranking genes, respectively. In contrast, under low-stringency AP conditions, the differences between the output of highly and lowly ranked genes were smaller. Notably, bottom-ranking neurons can be activated in real neural networks when the neurons in the preceding layer are inhibitory neurons. Although each layer of the GMN can only function as a high or low pass filter, the GMN can have various functions when layers with different APs are combined, as shown in Fig. 5 and Supplementary Fig. 4. Notably, the GMN can be readily connected either in series or in parallel by connecting neurons according to their gene expression, without adding any learning steps. Our results with the GMN suggest that a layer or a set of layers can function as an information-processing element, while in most artificial neural networks, single neurons are considered to be the primary information-processing elements. It is noteworthy that neurons in the GMN can also be used as a processing unit, even in deep layers, since the response specificity was still sharp in deep layers.

Collective network information is usually represented by a list of activated nodes, which is equivalent to the active-neuron array. However, the gene code can also be used to represent network information that is associated with overlapping densely connected subnetworks. The densely connected subnetworks of community structures^25^ were demonstrated to have strong effects on certain network dynamics, such as the spread of infectious diseases^26^. Highly connected subnetworks are frequently found in the real world such as social and biological networks being two examples. Notably, any network with densely connected clusters can potentially be represented and analyzed as a GMN by assigning a gene to every densely connected subnetwork. If such networks were analyzed as GMNs, active persons or neurons would be represented by the neuron code, while the activity of communities or heavily connected neuronal groups would be represented by the gene code. When subnetworks overlap, separating them into discrete subnetworks may not be productive for information analysis. With the GMN, heavily overlapping subnetworks can be analyzed without separating the subnetworks, using the information coding of the active gene array. Thus, the GMN may be a new tool for analyzing the information flow in networks with overlapping, heavily connected subnetworks.

Many biological networks, such as gene regulatory networks and metabolic protein networks, contain heavily connected subnetworks^25^,^27^,^28^,^29^,^30^. In such networks, if genes could be assigned to each subnetwork, the state of a network varying according to inputs could be described by the active-gene array. We found that the partial loss or a flaw in the connections of the GMN did not affect the reliability of information in the network. This feature of the GMN is beneficial for analyzing real-world networks such as biological networks, which are impossible to describe completely, and in which the heavily connected areas are not complete.

Notably, the architecture of the circular GMN is equivalent to the one-mode projection of bipartite networks, such as the movie-actor network and the collaboration network^31^. In the movie-actor network, “Movie titles” corresponds to genes and “Actors” corresponds to neurons. The information flow in such networks may be traced using active-gene arrays.

In the brain, information transfer could be represented by the activity of heavily connected neuronal groups or by simultaneously activated neuronal groups, the combination of which changes in response to environmental and/or behavior changes. Interestingly, this notion fits well with the cell-assembly theory of neural information coding, which proposes that neural information is encoded by the combination of active neuron groups that overlap one another^15^,^32^,^33^. In the GMN, the active-gene array represents the relative activity among overlapping subnetworks. Thus, use of the active-gene array may contribute to the decoding of neural information.

Microcircuits in the brain are rich in bidirectional connections and clusters^34^,^35^,^36^,^37^, suggesting that normally functioning neuronal networks exhibit features similar to the GMN. Regarding the real genes corresponding to the “genes” in the GMN, the clustered protocadherins (cPcdhs), of which there are 58 members^38^,^39^, are among the most plausible candidates. Each neuron expresses 5 to 10 of the 58 members in a random manner^40^,^41^. The cPcdhs promote cell-cell adhesion such that only cells expressing the same cPcdh member adhere to each other^42^,^43^. In the present study, genes were randomly expressed by neurons in the GMN. The reliable transfer and consistent transformation of information by the GMN was based on the nonbiased, yet overlapping distribution of genes, generated as a consequence of random expression. Interestingly, the random gene expression generates an enormous variety of neurons, known as neuronal diversity, at the neuron level, despite the nonbiased uniformity in the total gene expression at the layer or network level.

In conclusion, we found that non-linear activation of the GMN resulted in the systematic processing of network information. This process could be explicitly described by a vector representing the expression of genes in active neurons. These findings may lead to new insights into the information processing by cluster-rich networks in the real world, such as neural networks and human populations, and provide a novel method for the prediction and control of collective network information.

## Methods

All of the computer experiments were carried out using MATLAB (Mathworks, MA).

### Circular GMNs

To analyze the architecture of the GMN, circular GMNs with undirected edges were used. Parameters such as neuron numbers, GR size, and the number of genes expressed in each neuron, were determined as described in the corresponding sections. In the GMN, a pair of neurons was connected when both expressed one or more common genes. The number of connections between a pair of neurons was equivalent to the number of shared genes unless otherwise mentioned.

For the calculations of the short path length and the clustering coefficient, multiple connections between any neuron pair were considered as a single connection.

In the experiments in which the effect of GR size was examined using GMNs with various GR sizes, the expressed genes were selected randomly from a gene repertoire and assigned to neurons one by one in a rotational manner until the number of connections in the network reached 500.

### Estimation of the reliability of information transfer in multi-layer GMNs

The faithfulness of information transfer was estimated by the cross-layer consistency of distances (L1-norm) between a pair of information inputs. The cross-layer consistency was assessed using the active-neuron array and the active-gene array. One thousand pairs of inputs were used for each network. The correlation between the distances of the same output pairs obtained from a preceding layer and the following layer was plotted (Supplementary Fig. 6). The slope obtained by linear regression analysis of the distances plotted was used as the score of reliability of a network.

In the experiments analyzing the effect of GR size on reliability, GMNs were constructed in which the number of genes expressed per neuron was set to 3, 5, 7, 22, or 71 with gene repertoires of 20, 50, 100, 1,000, or 10,000 genes, respectively. The number of connections formed between the layers of these GMNs was 450,000, 500,000, 512,000, 484,000, and 504,100, respectively.

### Signal transmission in complex GMNs

The transmission in the GMN has polarity, positive or negative. When the transmission polarity was positive, the input vector was used as is. When the transmission polarity was negative, which corresponded to the situation where the preceding layer was comprised of inhibitory neurons, the output vector was multiplied by −1. When two or more preceding layers were connected to a subsequent layer in a complex GMN, such as those shown in Fig. 5d,e, the resulting input vectors in the subsequent layer were mixed at a ratio called the combination ratio.

## Additional Information

**How to cite this article**: Kitsukawa, T. and Yagi, T. The transfer and transformation of collective network information in gene-matched networks. *Sci. Rep*. **5**, 14984; doi: 10.1038/srep14984 (2015).

## Supplementary Material

Supplementary Information
