# Supplementary material for: The transfer and transformation of collective network information in gene-matched networks
Source: Sci Rep. 2015 Oct 9;5:14984. doi: 10.1038/srep14984 (PMC4598864; doi:10.1038/srep14984)
Supplement: Supplementary Information [file srep14984-s1.pdf]

The transfer and transformation of collective network information in gene-matched networks

Authors: Takashi Kitsukawa and Takeshi Yagi

Supplementary Figures and legends

Supplementary Methods

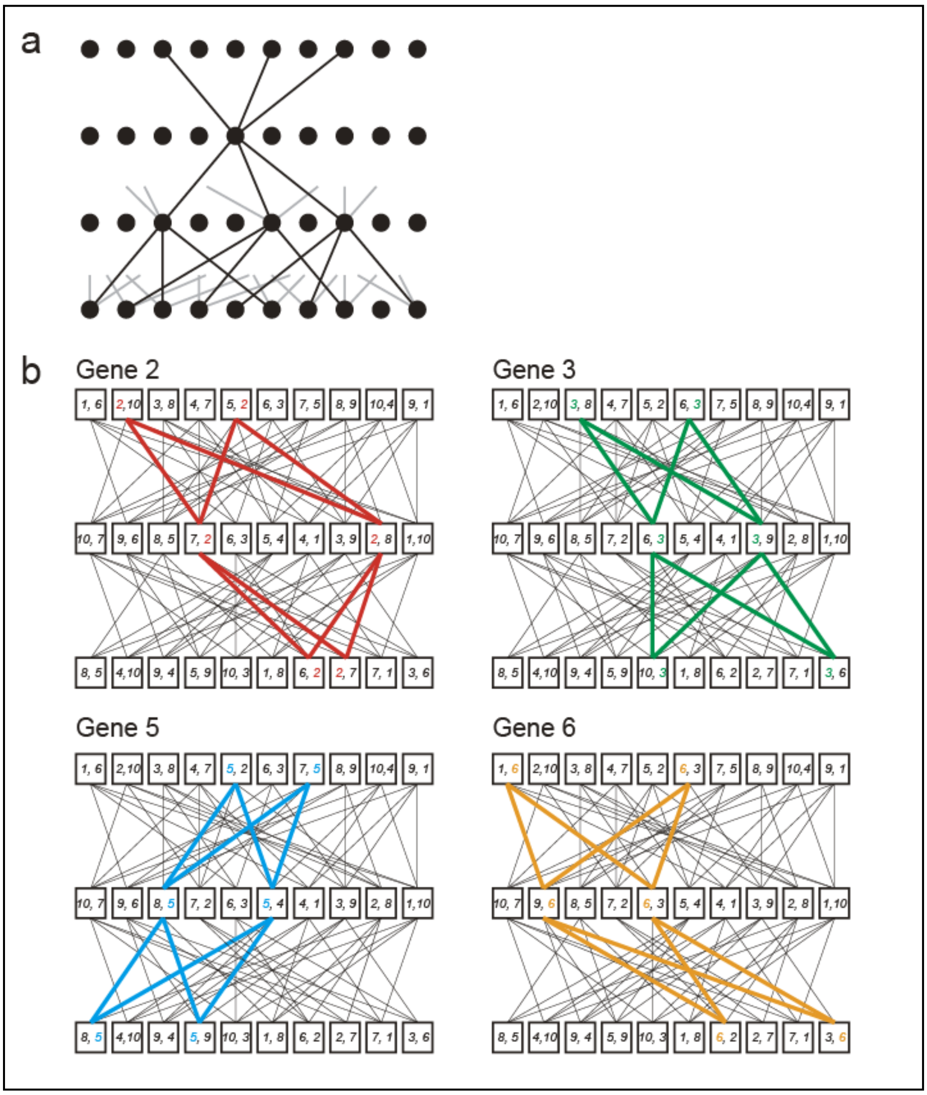

Supplementary Figure 1 | Controlled divergence of signals by genes expressed in the GMN. a, Averaging effect of the random network. Black closed circles represent neurons. The random convergence and divergence of signals is equivalent to averaging. **b**, Complete sub-networks in a three-layer GMN in which each neuron expresses two genes selected from a GR of 10 genes. The subnetworks composed of neurons expressing genes *2*, *3*, *5* and *6* are highlighted in red, green, blue, and yellow, respectively. Each subnetwork is complete, suggesting that the information within a subnetwork will be retained across the layers. Thus, the information content of the entire network may be divided and stored in subnetworks.

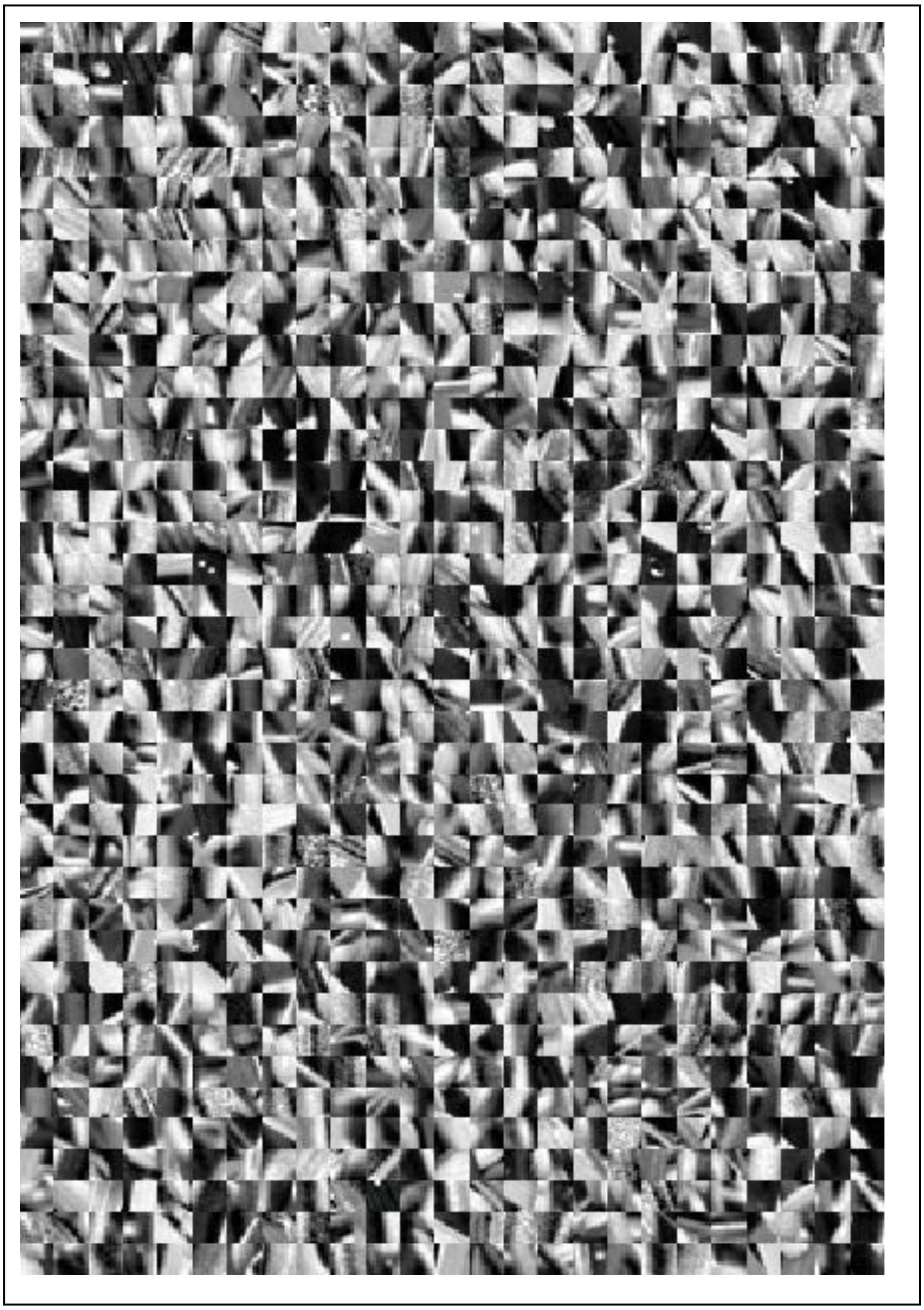

Supplementary Figure 2 | 1000 samples of 12×12 pixel images randomly selected from the original photograph in Fig. 4a.

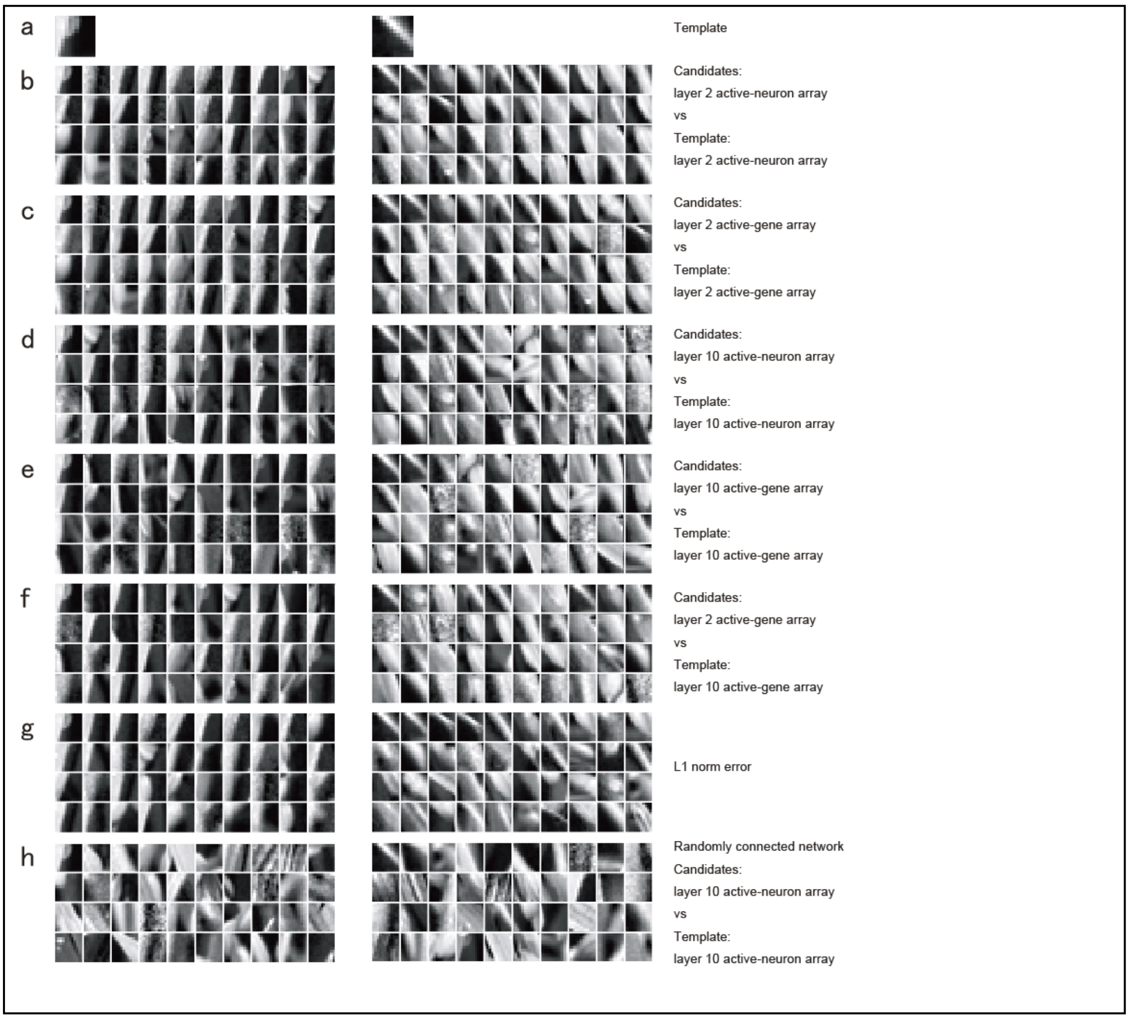

Supplementary Figure3 | Images listed according to their similarity to the active-neuron or active-gene arrays. Randomly chosen images (candidates, Supplementary Fig. 2) from the photograph in Fig. 4a were used as inputs in a 10-layer GMN. The active-neuron arrays or active-gene arrays of these images were compared to those of the two template images (a). **b**, Top 40 images showing similarity between the active-neuron arrays of layer 2 and both the layer 2 candidate and template images. **c**, Top 40 images showing similarity between the active-gene arrays of layer 2 and both the layer 2 candidate and template images. **d**, Top 40 images showing similarity between the active-neuron arrays of layer 10 and both the layer 10 candidate and template images. **e**, Top 40 images showing similarity between the active-gene arrays of layer 10 and both the layer 10 candidate and template images. **f**, Top 40 images showing similarity between the active-gene arrays of layer 10 and both the layer 2 candidate and template images. **g**, Top 40 images listed according to the least square error. **h**, Top 40 images showing similarity between the active-neuron array of layer 10 and the layer 10 candidate images in a random network.

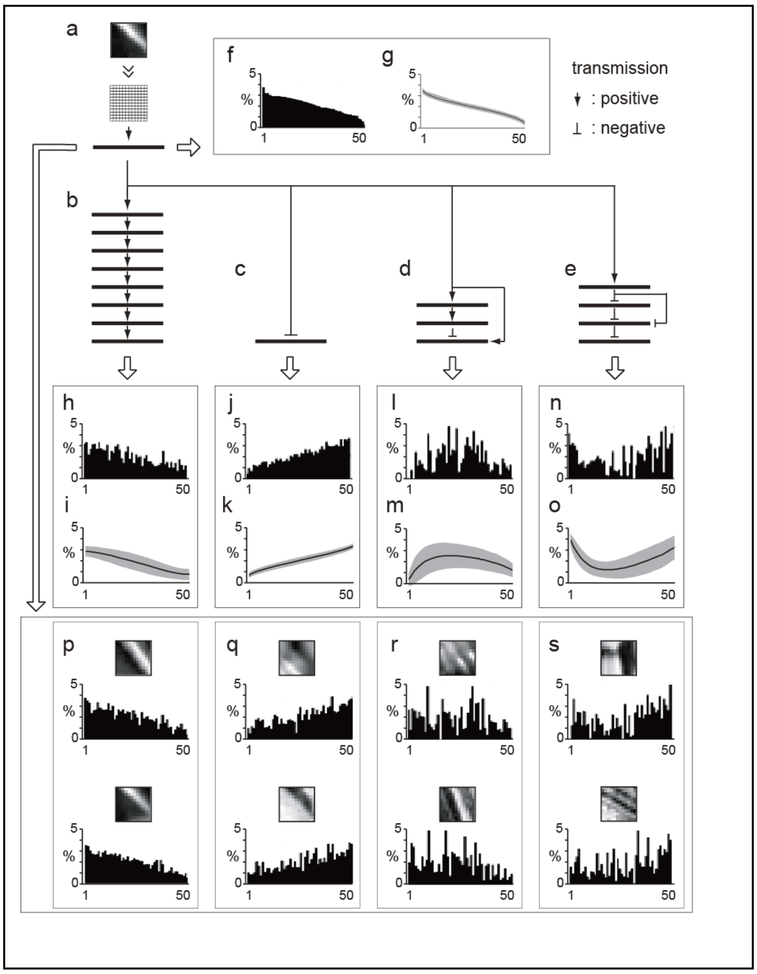

Supplementary Figure 4 | Another example of the transformation of the active-gene arrays by various GMNs. The GMNs of GR = 50 and GE =5 were used. The transmission polarities are specified with arrows (positive) and bars (negative). **a**, template image and the common part (layer 1 and 2). AP(layer2) = 50%. **b**, AP(layers 3~10) = 50%. **c**, AP(layer 3) = 50%. **d**, The GMN designed after the cerebellar network with layer 2 to 5 corresponding to the pontine nuclei, granule cell layer, Purkinje cell layer and cerebellar nuclei, respectively. AP(layer 3~5) = 25%. The combination ratio between main and side branches was 1:2. **e**, The GMN designed after the basal ganglia with layer 3 to 6 corresponding to the striatum, external glubus pallidus, substantia nigra pars reticulata and thalamic nuclei, respectively. AP(layers 3, 4, 5, 6) = 25, 10, 25, 25%. The combination ratio between main and side branches was 1:2. **f**, the active-gene array of layer 2 aligned in descending order by the template image in a. The gene order in this re-aligned array is called the input order. **g**, the active-gene array in layer 2 averaged over 5000 candidate images (sd, gray area). **h**, **j**, **l**, **n**, the active-gene arrays at the last layer of the GMNs b, c, d and e, respectively, activated by the template image a. **i**, **k**, **m**, **o**, the active-gene arrays averaged over the 5000 images at the last layer of the GMNs b, c, d and e, respectively (average, solid line; sd, gray area). **p**, **q**, **r**, **s**, the example images listed according to the similarity of their active-gene arrays at layer 2 to the active-gene array of the template image at the last layers of GMNs b,c,d and e, respectively. The active-gene arrays shown in h-s are realigned in the input order.

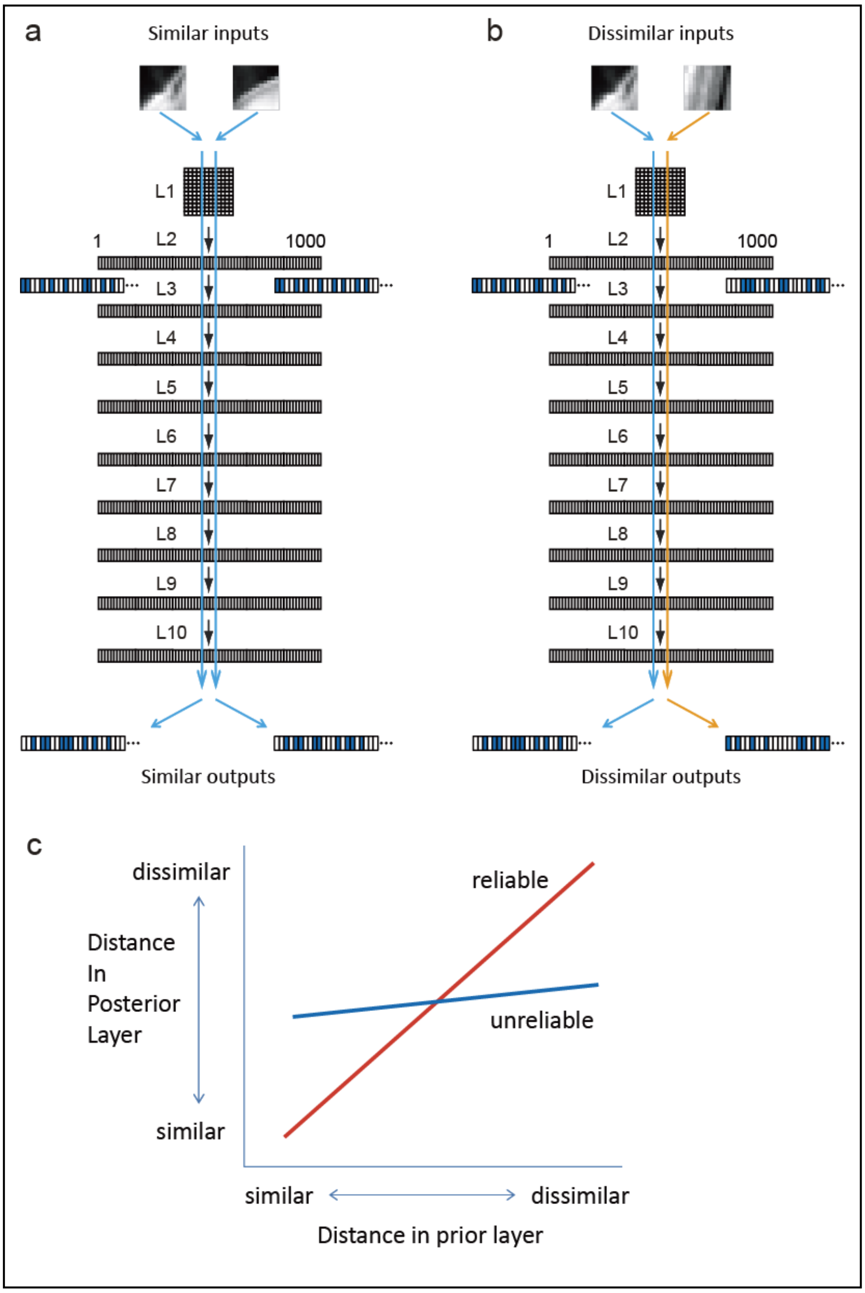

Supplementary Figure 5 | Cross-layer consistency: Analysis of similarity and distance between a pair of inputs and outputs.
a, **b**, If a network demonstrates reliable information transfer, similar inputs should return similar outputs (a), and dissimilar inputs should return dissimilar outputs (b), even after passing through multiple layers. In addition, the distance between a pair of independent inputs should correlate with the distance of their outputs. **c**, When these relationships between the input and output pairs are plotted, the slope of the data points should be close to "1" for a reliable network. In contrast, if a network is not reliable, the slope will be flat.

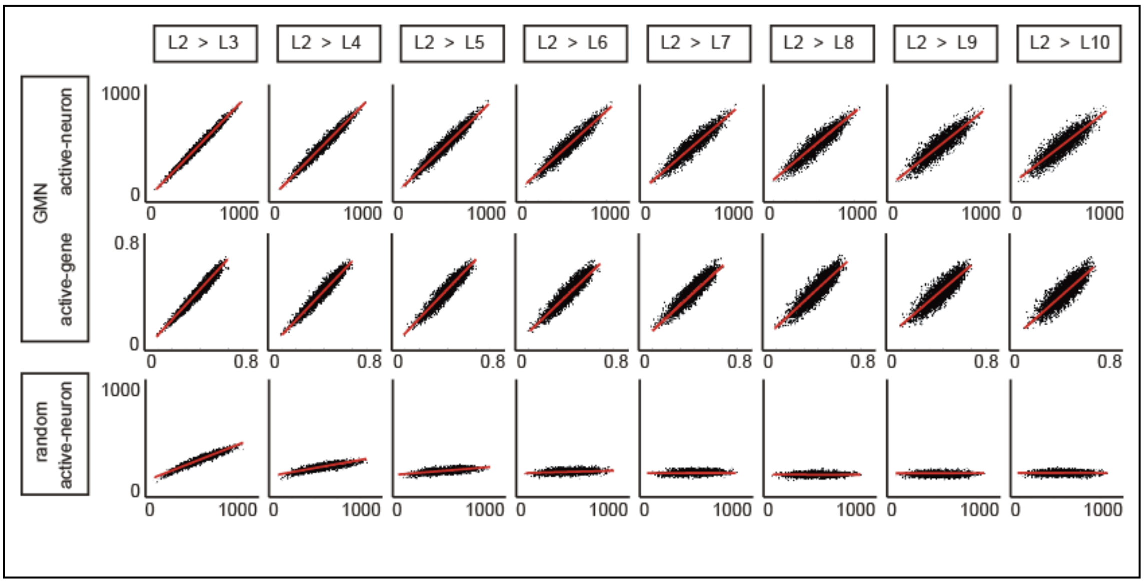

Supplementary Figure 6 | Analysis of cross-layer consistency. One thousand pairs of images were used as inputs in 10-layer networks. The distance between a pair of outputs at layers 3 to 10 (from left to right) was plotted against the distance between the corresponding pair of outputs at layer 2 (outputs at layer 2 were used as inputs in layer 3). The distances were calculated using the active-neuron (top) and active-gene arrays (middle) of GMNs (GR=50, GE=5), and the active-neuron arrays of random networks (bottom). Red lines represent the linear approximation of distance distribution obtained by linear regression analysis.

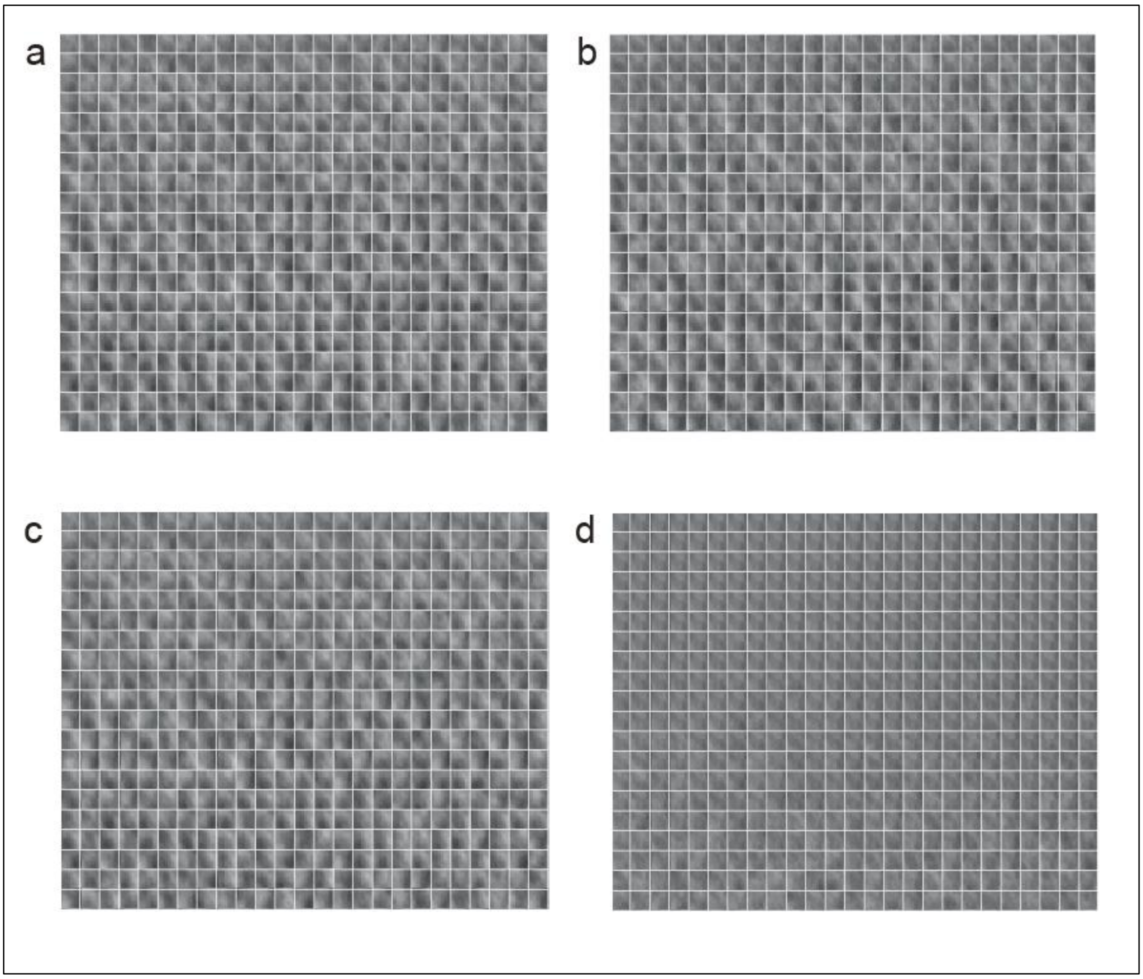

Supplementary Figure 7 | Averaged input images for individual neurons. The averaged images of the top 500 neurons most frequently activated in a layer (1000 neurons) are shown (top left to right bottom). **a**, The third layer of a GMN of 1000 neurons (GR=50, GE=5, AP=50%). **b**, The tenth layer of the same GMN. **c**, The second layer of a random network. **d**, The tenth layer of the same random network.

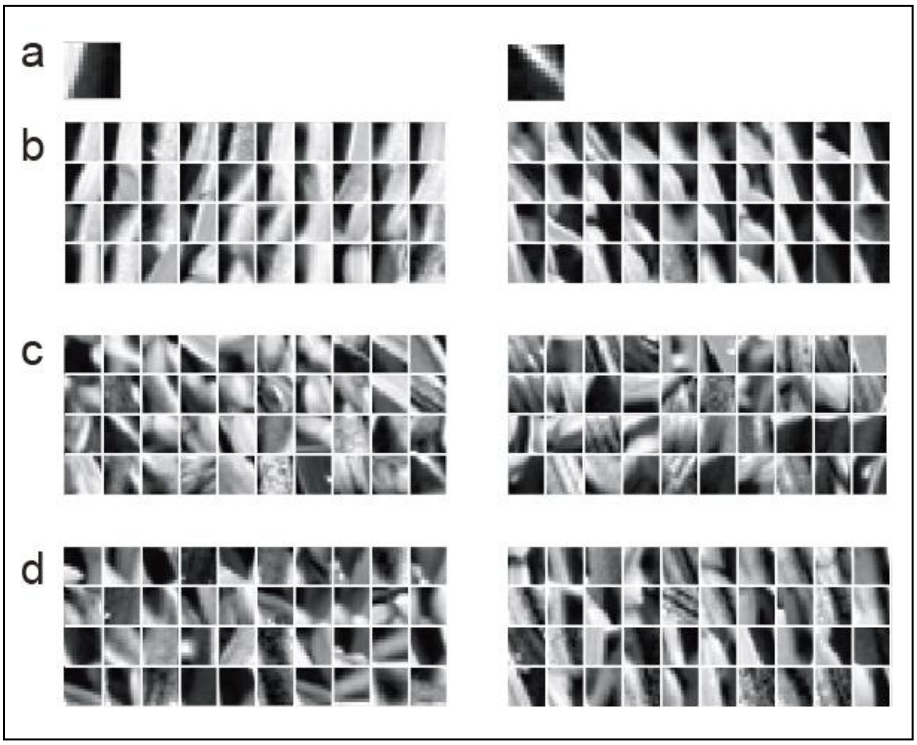

Supplementary Figure 8 | Images corresponding to the active-gene arrays obtained from complex GMNs. a, Template images. The active-gene arrays of these images, obtained from the last layers of each complex network, were used as templates. The images, which showed similar active-gene arrays at layer 2 to the templates, are shown in b–d. **b,** Top 40 images identified when bottom-ranked neurons were activated by the GMN shown in Fig 5c. **c**, Top 40 images identified when the complex GMN shown in Fig. 5d was used. **d**, Top 40 images identified when the complex GMN shown in Fig. 5e was used.

Supplementary methods

Circular GMNs

Circular GMNs were established using the gene expression matrix, **E** and the connection matrix, **C**. The gene expression matrix **E**, which is an $N \times Gr$ matrix, describes the gene expression, where N is the number of neurons and Gr is the GR size. The component of **E**, e_{ij}, describes the number of jth genes expressed in the ith neuron.

$$e_{ij} = \begin{cases} \text{n}, & \text{when neuron } i \text{ expresses } n \text{ molecules of gene } j, \\ 0, & \text{when neuron } i \text{ does not express gene } j. \end{cases}$$

The connection among the neurons in the circular GMN is described by an $N \times N$ matrix **C**,

$$\mathbf{C} = \mathbf{E}\mathbf{E}^{\mathbf{T}} - \mathbf{D}$$

where **D** is an $N \times N$ diagonal matrix, whose diagonal components are equal to those of $\mathbf{E}\mathbf{E}^{\mathbf{T}}$. The matrix **D** was subtracted so that the diagonal components of **C** would be zero, because the GMN does not possess self-edges. The superscript T denotes the matrix transpose operation.

When the short path length and the clustering coefficient were calculated, multiple connections between any neuron pairs were considered as a single connection. The clustering coefficient CC was calculated as

$$\text{CC} = \frac{1}{N}\sum_{N} \frac{\text{number of triangles}}{\text{number of adjacent node pairs}}.$$

In the experiments in which the effect of the GR size was examined using GMNs with various GR sizes, expressed genes were selected randomly from a gene repertoire and assigned to neurons one by one in a rotational manner until the number of connections in the network reached 500.

Multi-layer GMN construction

A layer of GMNs was formed with 1,000 neurons unless otherwise mentioned. All connections were made between neighboring layers. No connection was made within a layer. Neuron pairs in adjacent layers were connected when both neurons expressed one or more common genes. The number of connections between a pair of neurons was equivalent to the number of shared genes. The multi-layer GMNs were directed networks, and signals were always transmitted from the top to the bottom layer. In all multi-layer GMNs, unless otherwise mentioned, neurons expressed 5 genes randomly selected from a repertoire of 50 genes.

To generate a connection matrix between two layers in the layered GMN, the

gene expression matrix of the nth layer, **E**(n), and $n+1^{th}$ layer, **E**(n+1), were concatenated to establish a gene expression matrix for the two layers, **E**(n,n+1).

$$\mathbf{E}_{(n,n+1)} = [\mathbf{E}_{(n)}, \mathbf{E}_{(n+1)}] = \begin{pmatrix} e^{n}_{1\,1} & \dots & e^{n}_{1\,Gr} \\ \dots & \dots & \dots \\ e^{n}_{N\,1} & \dots & e^{n}_{N\,Gr} \\ e^{n+1}_{1\,1} & \dots & e^{n+1}_{1\,Gr} \\ \dots & \dots & \dots \\ e^{n+1}_{N\,1} & \dots & e^{n+1}_{N\,Gr} \end{pmatrix}$$

Thus, **E**(n,n+1) is a $2N \times Gr$ matrix where N is the number of neurons and Gr is the gene repertoire. The multiplication of this matrix yields a $2N \times 2N$ matrix **C**, which can be partitioned into four $N \times N$ matrices as follows.

$$\mathbf{C} = \mathbf{E}_{(n,n+1)}\,\mathbf{E}^{T}_{(n,n+1)} = \begin{bmatrix} \mathbf{C}_{(n,n)} & \mathbf{C}_{(n+1,n)} \\ \mathbf{C}_{(n,n+1)} & \mathbf{C}_{(n+1,n+1)} \end{bmatrix}$$

$\mathbf{C}_{(n,n+1)}$ was used for the connection matrix from the nth layer to $n+1^{th}$ layer.

Information transfer experiments using multi-layer GMNs

Five thousand images (12×12 pixels) were randomly collected from a photograph (Fig. 4a and Supplementary Fig. 2) and used as inputs to the multi-layer network. The 12×12 images were reshaped into a 144 dimensional vector **x**. $\mathbf{x}=(x_1, \dots, x_{144})^T$ captures the pixel intensity in the image (256 gradations of black to white). The connection matrix between layers 1 and 2, $\mathbf{C}_{(1,2)}$, is a 1000×144 matrix. The input to the second layer, $\mathbf{I}_{L2}$ was calculated as

$$\mathbf{i}_{L2} = \mathbf{C}_{(1,2)}\,\mathbf{x}$$

In other words, the sum of inputs for i^{th} neuron in the second layer was calculated as

$$\mathbf{i}_{\mathbf{L2}}(i) = \sum_{j=1}^{144} \mathbf{x}(j)\; c_{ij}$$

where c_{ij} is the component in the ith row and j^{th} column of $\mathbf{C}_{(1,2)}$, which represents the number of connections between the jth neuron in the first layer and the ith neuron in the second layer (c_{ij} is '0' where no connection was formed). Thus, $\mathbf{i}_{L2}$ corresponds to the sum of inputs to each neuron in layer 2 (see Fig. 3a). The activated neurons in the second layer were determined according to the amount of input. The percentage of activated neurons, the activation percentage (AP), was set for each layer. In the information transfer experiment, neurons ranking in the upper 50% in the level of input received (represented as AP50), were activated in layers 2 to 10. The output array, which represents the activity of neurons in a layer, consists of as many components as the number of neurons in the layer, in which '1' stands for activated neurons and '0' for non-activated neurons. Thus, the output array for layer 2 is

$$\mathbf{o}_{\mathrm{L2}}(k) = \begin{cases} 1 & , if\, \mathbf{i}_{\mathbf{L2}}(k) \text{ in AP} \\ 0 & , otherwise \end{cases}.$$

Then, the $\mathbf{o}_{\mathrm{L2}}$ is transferred to layer 3 as

$$\mathbf{i}_{\mathrm{L3}} = \mathbf{C}_{(2,3)}\ \mathbf{o}_{\mathrm{L2}}$$

and then $\mathbf{o}_{\mathrm{L3}}$ is determined according to the AP for layer 3. In this manner, signals were transferred to subsequent layers. The output array **o** is also called the active-neuron array.

In the GMN, output was also analyzed using the active-gene array. The active-gene array represents the profile of genes expressed in activated neurons in a particular layer. The active-gene array **g** is thus comprised of as many components as the GR size, $\mathbf{g} = (g_1, \ldots, g_{Gr})^{\mathrm{T}}$, where *Gr* is the GR size. g_{n} captures the number of the nth gene expressed in activated neurons. The active-gene array **g** was calculated with the gene expression matrix and the output array **o** (the active-neuron array). For example, the active-gene array for the output in layer 3, $\mathbf{g}_{\mathrm{L3}}$, was calculated as

$$\mathbf{g}_{\mathrm{L3}} = \mathbf{E}_{\mathrm{L3}}^{\mathrm{T}}\ \mathbf{o}_{\mathrm{L3}}$$

Estimation of reliability in information transfer

The faithfulness of information transfer was estimated by the cross-layer consistency of distances between a pair of information inputs. The cross-layer consistency was assessed using 1) the active-neuron array **o** and 2) the active-gene array **g**. When the information is transferred faithfully across layers, similar outputs from a preceding layer should result in similar outputs of the following layer (Supplementary Fig. 5). More specifically, the distance between outputs in a preceding layer should positively correlate with the distance between resulting outputs in the following layer.

1) Estimation of faithfulness in information transfer using the active-neuron array

The estimation of faithfulness with the active-neuron array was carried out using the distance between two different outputs. The distance between two active-neuron array pairs (D_{neuron}), $\mathbf{o}^1$ and $\mathbf{o}^2$, was calculated as

$$D_{neuron} = \sum_{n=1}^{N} \left| \mathbf{o}^1(\mathrm{n}) - \mathbf{o}^2(\mathrm{n}) \right|$$

where *N* is the number of neurons in the layer. One thousand pairs of outputs were used for each network. The correlation between the distances of the same output pairs obtained from a preceding layer and the following layer is plotted in Supplementary Fig. 6, in which the horizontal axis indicates the distance in the preceding layer and the vertical axis indicates the distance in the following layer. The slope obtained by linear

regression analysis of the distances was used as the score of reliability of a network. The slopes obtained from GMNs and degree-matched random networks were compared.

2) Estimation of information transfer using expressed genes

The estimation of faithfulness using genes expressed in active neurons was carried out using the distances (D_{gene}) between a pair of active-gene arrays, $\mathbf{g}^1$ and $\mathbf{g}^2$.

$$D_{gene} = \sum_{n=1}^{Gr} \left| \mathbf{g}^1(\mathrm{n}) - \mathbf{g}^2(\mathrm{n}) \right|$$

where *Gr* is the GR size. The slope obtained by linear regression analysis of the distances was used for the score of network reliability as in (1).

Image search

Five thousand images (12×12 pixels) randomly chosen from a photograph (Fig. 4a) were used as candidate images. The information of the template images (Fig. 5a and Supplementary Fig. 4a) was transferred and transformed through the multi-layer networks shown in Fig. 5b-e. The active-gene arrays of the template images obtained in the last layer of each GMN were compared to the active-gene arrays of the candidate images obtained in layer 2. The difference between the active-gene arrays of template and candidate images was calculated in the same manner as the calculation of D_{gene}.
